# Current News

**Published:** 1895-02

**Authors:** 


### Current News.

---

### SOUVENIR VOLUME OF THE WELLS CELEBRATION.

THE chairman of the Executive Committee of the Horace Wells Fiftieth Anniversary Celebration announces that the papers read by Professors Fillebrown and Garretson at the meeting, and the speeches delivered at the banquet have been prepared for publication in the proposed souvenir volume, and will be issued upon the receipt of a sufficient number of subscriptions to cover the expense. Price $1.50. The undersigned will receive subscriptions, receipt for same, and deliver the book upon completion.

J. D. THOMAS,
*Chairman.*

912 WALNUT STREET, PHILADELPHIA.

